# Supplementary figures and images for: The Epidemiologic Transition in French Guiana: Secular Trends and Setbacks, and Comparisons with Continental France and South American Countries
Source: Trop Med Infect Dis. 2023 Apr 8;8(4):219. doi: 10.3390/tropicalmed8040219 (PMC10143289; doi:10.3390/tropicalmed8040219)

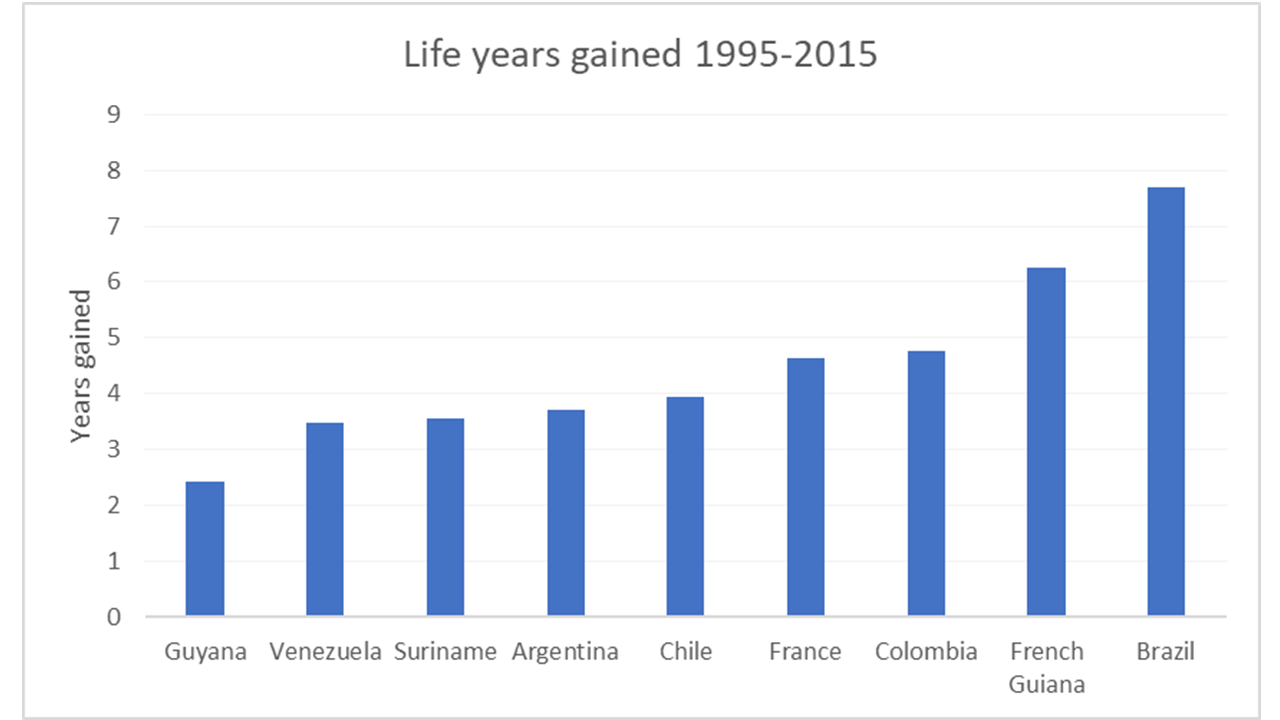

Supplement: Supplementary file 1 [file tropicalmed-08-00219-s001.zip › Supplementary figure S1.tif]

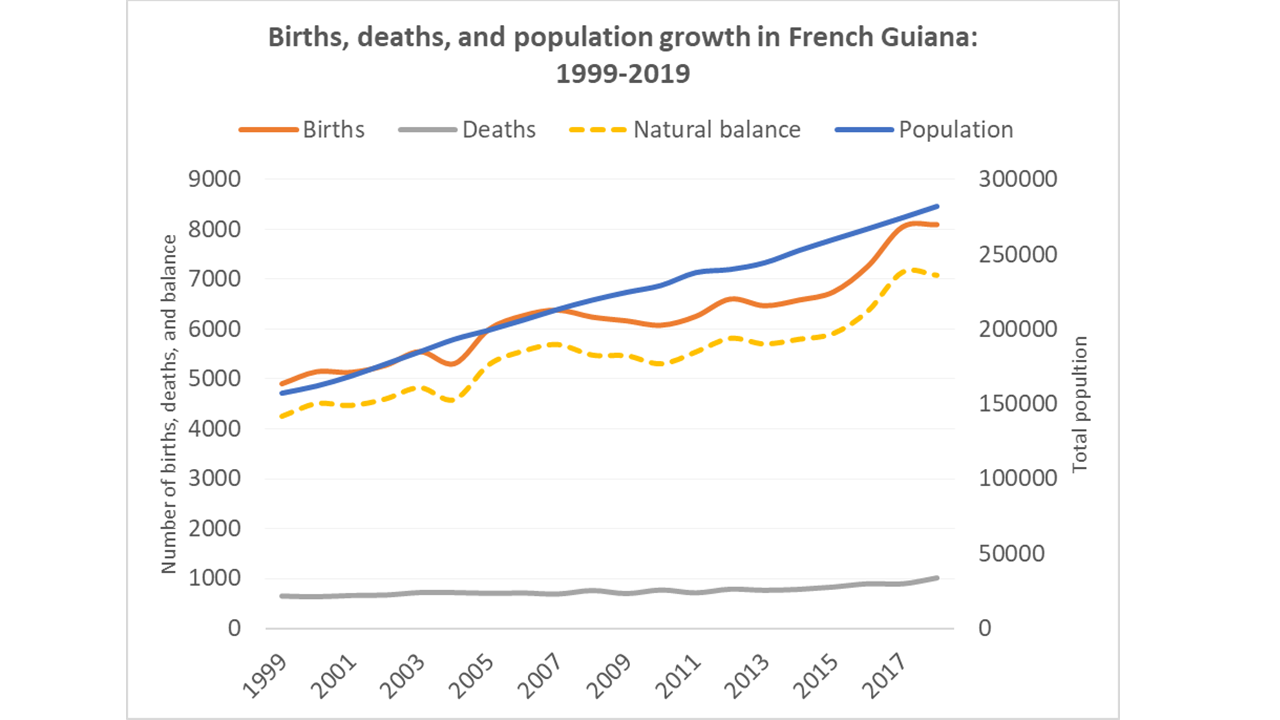

Supplement: Supplementary file 1 [file tropicalmed-08-00219-s001.zip › Supplementary figure S2.tif]

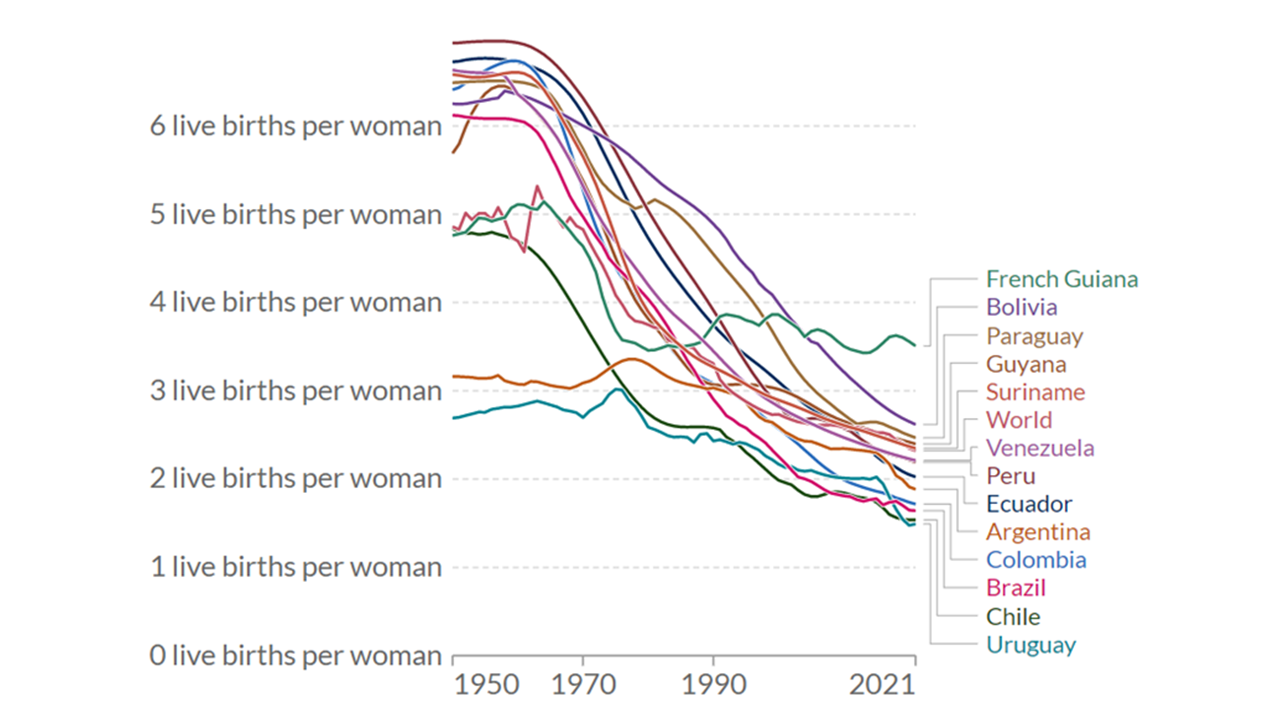

Supplement: Supplementary file 1 [file tropicalmed-08-00219-s001.zip › Supplementary figure S3.tif]

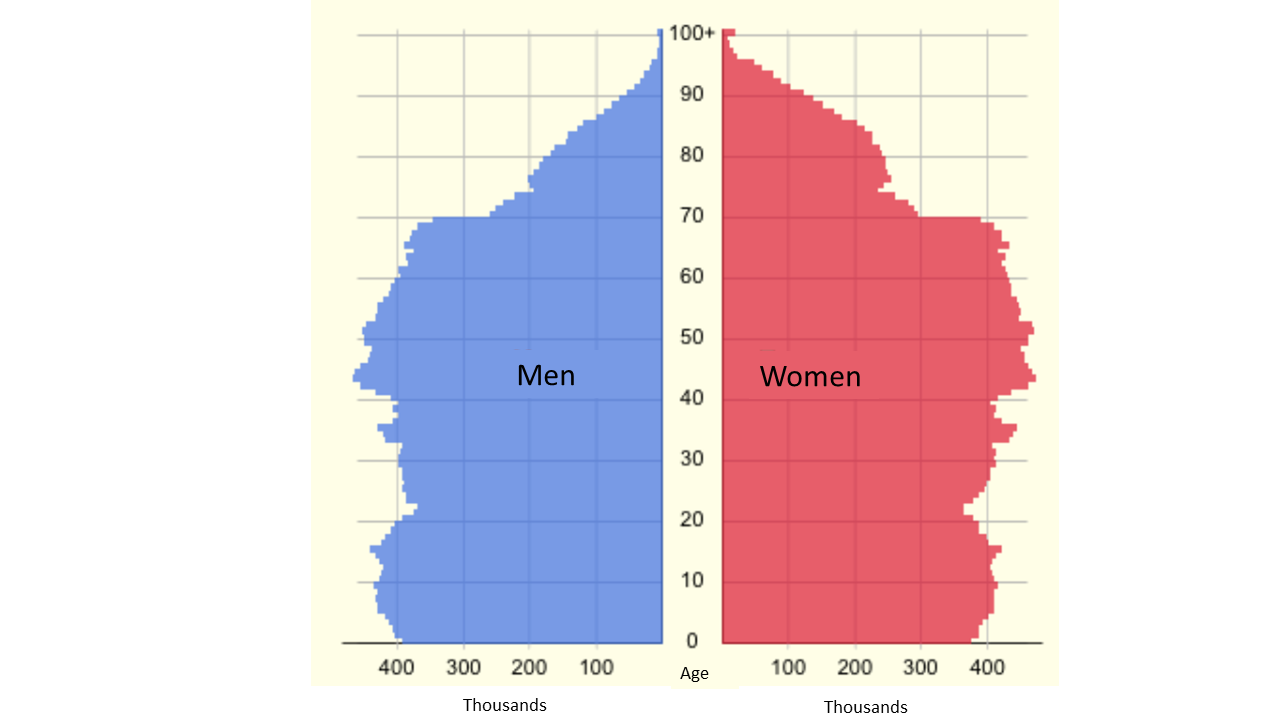

Supplement: Supplementary file 1 [file tropicalmed-08-00219-s001.zip › Supplementary figure S4.tif]
